# Implementation of Chatbot Technology in Health Care: Protocol for a Bibliometric Analysis

**DOI:** 10.2196/54349

**Published:** 2024-02-15

**Authors:** Zhao Ni, Mary L Peng, Vimala Balakrishnan, Vincent Tee, Iskandar Azwa, Rumana Saifi, LaRon E Nelson, David Vlahov, Frederick L Altice

**Affiliations:** 1 School of Nursing Yale University Orange, CT United States; 2 Center for Interdisciplinary Research on AIDS Yale University New Haven, CT United States; 3 Department of Global Health and Social Medicine Harvard Medical School Harvard University Boston, MA United States; 4 Department of Information Systems Faculty of Computer Science and Information Technology Unversity of Malaya Kuala Lumpur Malaysia; 5 Centre of Excellence for Research in AIDS Faculty of Medicine University of Malaya Kuala Lumpur Malaysia; 6 Infectious Disease Unit Faculty of Medicine University of Malaya Kuala Lumpur Malaysia; 7 Section of Infectious Disease Department of Internal Medicine Yale School of Medicine New Haven, CT United States; 8 Division of Epidemiology of Microbial Diseases Yale School of Public Health New Haven, CT United States

**Keywords:** artificial intelligence, AI, bibliometric analysis, chatbots, health care, health promotion

## Abstract

**Background:**

Chatbots have the potential to increase people’s access to quality health care. However, the implementation of chatbot technology in the health care system is unclear due to the scarce analysis of publications on the adoption of chatbot in health and medical settings.

**Objective:**

This paper presents a protocol of a bibliometric analysis aimed at offering the public insights into the current state and emerging trends in research related to the use of chatbot technology for promoting health.

**Methods:**

In this bibliometric analysis, we will select published papers from the databases of CINAHL, IEEE Xplore, PubMed, Scopus, and Web of Science that pertain to chatbot technology and its applications in health care. Our search strategy includes keywords such as “chatbot,” “virtual agent,” “virtual assistant,” “conversational agent,” “conversational AI,” “interactive agent,” “health,” and “healthcare.” Five researchers who are AI engineers and clinicians will independently review the titles and abstracts of selected papers to determine their eligibility for a full-text review. The corresponding author (ZN) will serve as a mediator to address any discrepancies and disputes among the 5 reviewers. Our analysis will encompass various publication patterns of chatbot research, including the number of annual publications, their geographic or institutional distribution, and the number of annual grants supporting chatbot research, and further summarize the methodologies used in the development of health-related chatbots, along with their features and applications in health care settings. Software tool VOSViewer (version 1.6.19; Leiden University) will be used to construct and visualize bibliometric networks.

**Results:**

The preparation for the bibliometric analysis began on December 3, 2021, when the research team started the process of familiarizing themselves with the software tools that may be used in this analysis, VOSViewer and CiteSpace, during which they consulted 3 librarians at the Yale University regarding search terms and tentative results. Tentative searches on the aforementioned databases yielded a total of 2340 papers. The official search phase started on July 27, 2023. Our goal is to complete the screening of papers and the analysis by February 15, 2024.

**Conclusions:**

Artificial intelligence chatbots, such as ChatGPT (OpenAI Inc), have sparked numerous discussions within the health care industry regarding their impact on human health. Chatbot technology holds substantial promise for advancing health care systems worldwide. However, developing a sophisticated chatbot capable of precise interaction with health care consumers, delivering personalized care, and providing accurate health-related information and knowledge remain considerable challenges. This bibliometric analysis seeks to fill the knowledge gap in the existing literature on health-related chatbots, entailing their applications, the software used in their development, and their preferred functionalities among users.

**International Registered Report Identifier (IRRID):**

PRR1-10.2196/54349

## Introduction

Chatbots are software applications that use computerized algorithms to simulate conversations with human users through text or voice interactions [1,2]. Since their inception in the 1960s, chatbots have found applications in various settings, such as airlines, banks, hotel chains, and information technology companies, serving as digital agents to handle and streamline customers’ queries and needs [3]. Compared to human agents, chatbots can efficiently respond to a large number of users simultaneously, conserving human effort and time while still providing users with a sense of human interaction [4]. Due to this advantageous feature, chatbots have been implemented in health care settings to automatically resolve or deflect repetitive calls, thereby reducing waiting times for health care consumers and enabling health care professionals to focus on more complex cases. Driven by the evolution of Industry 4.0, characterized by the integration of digital technologies, data, and automation to create more efficient and responsive societal systems, the future of high-quality health care hinges on the automation and digitalization of data exchange. Against this social-technological backdrop, artificial intelligence (AI) chatbots, also known as conversational AI, hold substantial promise as innovative tools for advancing our health care systems [5].

AI chatbots have been developed to automate and streamline various tasks for health care consumers, including retrieving health information, providing digital health support, and offering therapeutic care [6]. The literature reveals that AI chatbots commonly fulfill roles such as assisting individuals in scheduling medical appointments, identifying health clinics, and providing health educational information [7,8]. Research has also shown that health care professionals, patients, and families exhibit favorable attitudes toward the use of chatbot technology to enhance health outcomes [7,9-12].

While AI chatbots hold considerable potential to drive significant advancements and improvements in health care [13,14], their application in health care is still in its early stages. A significant barrier to the deployment of chatbot technology in health care systems is the lack of sophisticated AI algorithms capable of facilitating precise and personalized human-chatbot interactions to meet the expectations of health care providers or fulfill the needs of health care consumers [11]. For instance, in the United States, health-related chatbots have been developed to monitor the health status of patients with chronic heart failure [15], screen for osteoporosis in menopausal women [16], and detect colorectal cancer in the general population [17]. However, their effectiveness in clinical trials was found to be limited when compared to health professional assessments. To fully realize the potential of chatbot technology in health care systems, more studies are needed to develop more sophisticated AI algorithms that are culturally tailored, theoretically informed, and trained based on clinical needs [18-21]. Creating such sophisticated AI chatbots presents a challenge for both health scientists and chatbot engineers, necessitating iterative collaboration between the 2 [22]. Specifically, after chatbot engineers develop a chatbot prototype, health scientists evaluate it and provide feedback for further refinement. Chatbot engineers then upgrade the chatbot, followed by health scientists testing the updated version, training it, and conducting further assessments. This iterative cycle can impose significant demands in terms of time and funding before a chatbot is equipped with the necessary knowledge and language skills to deliver precise responses to its users.

Bibliometric analysis is a quantitative research method to discern publication patterns within a specific timeframe [23]. Scholars use this type of analysis to elucidate the intellectual structure of a particular area within the realm of existing literature [24]. Despite the increasing popularity of health-related chatbots, no bibliometric analysis has been conducted to examine their application. Studies on the coverage of health-related chatbot research have predominantly been conducted in the form of scoping or systematic reviews [19,25,26]. The current body of research papers lacks the breadth of a comprehensive scientific performance mapping analysis. Hence, this bibliometric analysis aims to identify the current status and emerging trends in chatbot technology research, serving as an initial stride for researchers worldwide to gain a comprehensive understanding on the landscape of health-related chatbots. This overview will facilitate the identification of areas for improvement and promote the integration of chatbot technology into health care systems.

Chatbot technology should be promoted in the health care system because many digital health interventions have proven effective but are not implemented in real clinical settings, as they often require high-intensity and sustained human inputs. For example, they often require researchers to regularly and manually send personalized reminders, provide real-time guidance, and initiate referrals [27,28]. To bring population-level effects, digital health intervention needs to be automating personalized messages, modifying them based on responses, and providing new outputs in real time [29]. AI chatbots have the potential to achieve these goals. For example, our previous formative research indicates a high level of acceptance toward the use of chatbot technology among vulnerable populations who are at high risk for HIV [2]. Additionally, we have conducted beta testing for chatbot technology in promoting HIV testing and prevention and found that participants believed chatbot technology provided them with a platform to protect their safety and privacy. This was particularly important in environments where stigma and discrimination toward HIV exist, and where same-sex behaviors are criminalized. Compared with the conventional health care use model, where people need to face stigma and discrimination from health care providers, chatbots can provide them with a safe platform to ask questions and receive consulting services. Therefore, promoting chatbot technology holds significance for enhancing the current health care system and an anonymous user setting in chatbots is necessary to protect health consumers’ privacy [2].

## Methods

### Bibliographic Search

We will conduct searches in the following databases: CINAHL (EBSCOHost), IEEE Xplore (IEEE), PubMed, Scopus (Elsevier), and Web of Science (Clarivate) to ensure a comprehensive coverage of research on health-related chatbots. We have identified a set of consensus search keywords related to chatbots and health care through a review of previous systematic reviews [18-21] and consultations with university librarians who possess expertise in informatics and digital health. Our search strategy includes keywords such as “chatbot,” “virtual agent,” “virtual assistant,” “conversational agent,” “conversational AI,” “interactive agent,” “health,” and “healthcare.” A retrieval search string, including the keywords with Boolean operators listed in Multimedia Appendix 1, will be used.

### Selection of Studies

We will include papers on chatbots that are used to promote health outcomes. All interventional and observational studies published as journal papers or conference proceedings will be included. To offer a holistic view of the evolving usage of chatbots in health care, we will not set restrictions on the year of publication. Moreover, we will not exclude papers published in non–English language to incorporate research findings from low- and middle-income countries [30]. Studies that do not discuss the use of chatbots to promote health or wellness will be excluded. Systematic reviews pertaining only to chatbot designs and development, purposes, or features will be excluded. Papers such as editorials, dissertations, preprints, and letters to the editor will also be excluded.

Search results from each database will be imported into Covidence (Veritas Health Innovation Ltd), a systematic review management software. Duplicate papers will be identified and removed. Five researchers will independently screen the titles and abstracts of all papers and categorize them as either “include,” “exclude,” or “unsure” based on the following inclusion criteria related to (1) chatbot and (2) health promotion. To provide a comprehensive overview of the current research on health-related chatbots, we will include papers about chatbots designed for various populations, including patients, clinicians, policy makers, or the general population. We will not exclude papers based on their study design. Papers marked as “unsure” will be downloaded and assessed for eligibility. The eligibility assessment will be performed by 2 authors (VB and VT) who are an AI consultant and a clinician. In the event of disagreements, the 2 authors will discuss in team meetings with the corresponding author (ZN) to reach a consensus.

### Data Extraction

Selected studies will be downloaded from Covidence and imported into VOSViewer (version 1.6.19; Leiden University), a Java-based bibliometric analysis visualization software application. We will use VOSViewer to analyze data related to chatbot publication patterns, encompassing the number of annual publications, the distribution of countries and institutions involved in chatbot research, the number of annual grants that supported chatbot research, the number of funders, the number of journals publishing chatbot research, the number of journal citations, the most prolific authors in the field of chatbots, author network maps, and the most frequently used keywords related to chatbots. Additionally, using Excel (Microsoft Corp), we will manually extract data regarding the number of interventional and observational studies, the methodologies used in creating chatbots, the number of chatbots deployed worldwide, and the usage of chatbots.

### Data Analysis

#### Research Characteristics

In this bibliometric analysis, we will analyze the characteristics of chatbot research based on the topics of the selected studies, identified through their reported keywords, such as primary functions and disease domains. We will report the frequency and percentage of the top keywords and topics by following the framework in previous research to measure the centrality of a keyword using its frequency scores [31].

#### Publication Patterns

We will report the trend of yearly number of publications and showcase the growth rate of publication by computing the monthly publication rate each year from the earliest publication selected to the latest. To control for the “publication noise,” a surge in publications following the releases of ChatGPT, we will stratify the data by publication date before and after the emergence of ChatGPT and conduct sensitivity analyses to distinguish between the direct impact of ChatGPT and other trends in chatbot research. This approach will help to ensure that our findings reflect the broader trends in chatbot technology research and are not disproportionately influenced by the recent increase in publications related to a single event or development. We will calculate the frequencies and percentages of publications for each journal and country in each publication year, whereby we identify the countries and institutions associated with the publication based on the affiliations of the corresponding authors. We will report the trend of research support by identifying the number of grants and funders as reported under each study’s source of funding. Moreover, we will compute the distribution of the following two indicators including (1) the methodology used to create chatbots and (2) the implementation of health chatbots.

#### Research Hot Spot and Connectedness

We will use the number of journal citations to construct bursts, whereby clusters will be sorted by the keywords used by the study. We will further report the most prolific authors based on a combined metric of the number of publications and citation frequency. We will present 2 author network maps using author names and research institutions affiliated with the listed authors. Authors and affiliated institutions will be the nodes within each network connected by edges representing the coauthorship of publications [32]. Edges will be weighted by the number of coauthorships between the same listed authors and their affiliated institutions.

### Ethical Considerations

This analysis does not involve recruiting human participants or providing interventions; therefore, ethical review and consent forms are not required. We hope that the findings from the manuscript will aid researchers, engineers, health professionals, funders, and policy makers in their future implementation of chatbot technology to facilitate innovative and efficient health care systems.

## Results

The preparation for the bibliometric analysis began on December 3, 2021, with the initial steps involving the research team familiarizing themselves with VOSViewer and CiteSpace, followed by digital consultations with 3 librarians from Yale University. Tentative searches in the databases yielded a total of 2340 papers. The official search phase began on July 27, 2023. Our goal is to complete the screening of papers and perform the analysis by February 15, 2024. We anticipate a significant increase in chatbot research following the emergence of ChatGPT.

## Discussion

AI chatbots hold strong potential to transform the field of health care. For example, ChatGPT, an AI chatbot developed by OpenAI, has sparked numerous discussions within the health care industry regarding the impact of AI chatbots on human health [13,14,33-38]. Our team has been developing an AI chatbot since June 2019, and we have discovered that developing a sophisticated AI chatbot capable of precise interaction with health care consumers, delivering personalized care, and providing accurate health-related information and knowledge remains a considerable challenge. One of the major obstacles faced by health scientists interested in chatbot research is their lack of familiarity with chatbots’ underlying technologies, such as the computational systems, software platforms, and underlying algorithms that train chatbots and enable their automation and individualization. Similarly, chatbot engineers may have limited insight into the challenges faced by patients and health care providers in real-world clinical settings, making it difficult for them to fully grasp the nuances and directions of chatbot technology. Such information asymmetry in interdisciplinary collaboration hinders health-advancing chatbot technology from reaching its full potential. More literature that can offer diverse stakeholders, ranging from health scientists, health care providers, to chatbot developers, insights into a holistic roadmap to creating effective chatbots to meet health care needs is thus urgently needed to facilitate such interdisciplinary endeavors.

Chatbot technology holds immense potential to enhance health care quality for both patients and professionals through streamlining administrative processes and assisting with assessment, diagnosis, and treatment. Used for health information acquisition, chatbot-powered search, as we anticipate, will become an important complement to traditional web-based searches. This trend is primarily driven by the convenience of chatbot-powered search for users, as it eliminates the need for users to manually sift through search results as required in traditional web-based searches. However, no recognized standards or guidelines have been established for creating health-related chatbots. We believe that with theory-informed and well-trained algorithms, chatbots can also be used as health care digital assistants to provide consumers and patients with quick, precise, and individualized answers. As demonstrated by the widespread adoption of ChatGPT in recent times, it is reasonable to anticipate that the development and implementation of AI chatbots will substantially enhance the efficiency and convenience of health care consumers’ information search. Existing studies have also shown that AI chatbots can increase health appointment volume by automating the scheduling process for health care consumers, achieved through a user-friendly platform that enables appointment scheduling, verification, and cancellation with ease, further leading to reduced no-show rates. For example, Weill Cornell Medicine reported a 47% increase in appointments booked digitally through the use of AI chatbots [39]. Moreover, chatbot technology can enhance the work efficiency of nurses, physicians, and other health care professionals by delivering prompt responses to inquiries related to clinical practice standards, eliminating the need to navigate prolonged websites or extensive clinical guidelines for specific information.

Despite the obvious benefits of chatbot technology in health care, several potential risks of using chatbots exist, including breaching privacy, providing misinformation, and generating systematically biased responses [2,7-9]. These risks are relevant to the nature of chatbot technology, in which chatbot developers need to maximize a personalized experience and enable chatbots to provide users with precision answers through training chatbots [12]. However, training chatbots requires chatbot technology to have access to a wealth of users’ personal data. This poses a major threat to using chatbots in health care. To address privacy issues, chatbot developers and researchers must ensure that users’ data are protected using encryption during human-chatbot interactions or when a chatbot needs to retrieve backend data [2]. Second, misinformation originates from the immature or flaws of the chatbot algorithms. Training a chatbot is an iterative process that demands a large data set and vetting of the outputs by researchers. During a chatbot creation, the earlier versions of the chatbot often provide redundant and impersonalized information that may prevent users from using the chatbot. To increase chatbot usability, a chatbot must be precise enough in its communications with users or can connect users to a human agent if necessary [11,12]. Third, even well-trained chatbots can provide biased responses or solutions to users [13]. This is particularly true if the data sets used to train chatbots are biased. To minimize these risks of using chatbots in health care, it is necessary for researchers to validate chatbot outputs and reduce biases in the data sets used to train a chatbot. Only by adopting this approach, quality chatbots with high usability can be used to promote health care.

To fully realize the potential of chatbot technology in improving health outcomes for everyone, sustained collaborative efforts from an interdisciplinary research team comprising chatbot engineers and health scientists are essential. To the best of our knowledge, this is the first study aimed at summarizing the current status and future trends of chatbots in the health care field. This study will provide a broad overview of publications on health-related chatbot research and bridge the knowledge gap in the existing literature, including software used for chatbot development, popular functionalities, and chatbots’ applications in health care. This study includes papers published since the inception of the chatbot and is not confined by the language of publication. Consequently, it offers a global perspective on the evolution of chatbots within the health care domain. One limitation of this study is its nature as a bibliometric analysis, which does not explore topics in the same depth as a systematic review.

## Appendix

Multimedia Appendix 1 A retrieval search string.

## Abbreviations

AI: artificial intelligence

## Data Availability

Data sharing is not applicable to this article as no data sets were generated or analyzed during this study.
