# Supplementary material for: Implementation of Chatbot Technology in Health Care: Protocol for a Bibliometric Analysis
Source: JMIR Res Protoc. 2024 Feb 15;13:e54349. doi: 10.2196/54349 (PMC10905346; doi:10.2196/54349)
Supplement: Multimedia Appendix 1 [file resprot_v13i1e54349_app1.docx]

Ovid MEDLINE(R) ALL

1 ((chat* or conversation* or voice) adj1 (bot or bots or assistant* or interface* or agent* or AI or automated)).mp.

2 (health* adj1 (bot or bots)).mp.

3 ((interactive or computer*) adj1 agent*).mp.

4 ((virtual or collaborative) adj1 (bot or bots or assistant*)).mp.

5 (dialogue system* or dialog system* or conversational system* or automated text* or automated telephone* or relational agent* or chatbot* or chatterbot* or teletriage*).mp.

6 (virtual adj (doctor* or physician* or nurse* or provider*)).mp.

7 ((intelligent or AI or computer* or virtual* or interactive*) adj3 triage).mp.

8 (izzy or superizzy or sensely or safedrugbot or woebot or abbi or florence beta or forksy or cancerchatbot or covidasha or gyant or ask-roo or eliza).ti,ab.

9 ((buoy or babylon or healthily) and (bot or bots or AI)).mp.

10 ((Natural Language Processing or NLP or Latent Semantic Analysis or Artificial Intelligence Markup Language or AIML or speech recognition) adj5 (bot or bots or assistant*)).mp.

11 or/1-10

Web of Science Core Collection

#1 TS=((chat* or conversation* or voice) near/1 (bot or bots or assistant* or interface* or agent* or AI or automated)) OR TS=(health* near/1 (bot or bots)) or TS=((interactive or computer*) near/1 agent*) OR TS=((virtual or collaborative) near/1 (bot or bots or assistant*)) or TS=("dialogue system*" or "dialog system*" or "conversational system*" or "automated text*" or "automated telephone*" or "relational agent*" or chatbot* or chatterbot* or teletriage*) OR TS=(virtual near/1 (doctor* or physician* or nurse* or provider*)) or TS=((intelligent or AI or computer* or virtual* or interactive*) near/3 triage) or TS=(izzy or superizzy or sensely or safedrugbot or woebot or abbi or "florence beta" or forksy or cancerchatbot or covidasha or gyant or ask-roo or eliza) or TS=((buoy or babylon or healthily) and (bot or bots or AI)) OR TS=(("Natural Language Processing" or NLP or "Latent Semantic Analysis" or "Artificial Intelligence Markup Language" or AIML or "speech recognition") near/5 (bot or bots or assistant*))

#2 TS=(medical or medicine or health* or disease* or illness* or patient* or hospital* or disorder*)

#3 #1 and #2

Scopus

( TITLE-ABS-KEY ( ( chat* OR conversation* OR voice ) W/1 ( bot OR bots OR assistant* OR interface* OR agent* OR ai OR automated ) ) OR TITLE-ABS-KEY ( health* W/1 ( bot OR bots ) ) OR TITLE-ABS-KEY ( ( interactive OR computer* ) W/1 agent* ) OR TITLE-ABS-KEY ( ( virtual OR collaborative ) W/1 ( bot OR bots OR assistant* ) ) OR TITLE-ABS-KEY ( "dialogue system*" OR "dialog system*" OR "conversational system*" OR "automated text*" OR "automated telephone*" OR "relational agent*" OR chatbot* OR chatterbot* OR teletriage** ) OR TITLE-ABS-KEY ( virtual W/1 ( doctor* OR physician* OR nurse* OR provider* ) ) OR TITLE-ABS-KEY ( ( intelligent OR ai OR computer* OR virtual* OR interactive* ) W/3 triage ) OR TITLE-ABS-KEY ( izzy OR superizzy OR sensely OR safedrugbot OR woebot OR abbi OR "florence beta" OR forksy OR cancerchatbot OR covidasha OR gyant OR ask-roo OR eliza ) OR TITLE-ABS-KEY ( ( buoy OR babylon OR healthily ) AND ( bot OR bots OR ai ) ) OR TITLE-ABS-KEY ( ( "Natural Language Processing" OR nlp OR "Latent Semantic Analysis" OR "Artificial Intelligence Markup Language" OR aiml OR "speech recognition" ) W/5 ( bot OR bots OR assistant* ) ) ) AND ( TITLE-ABS-KEY ( medical OR medicine OR health* OR disease* OR illness* OR patient* OR hospital* OR disorder* ) )

CINAHL

(chatbot* or “chat bot*” or “conversational agent*” or “health* bot*”)

IEEE Explorer

Chatbot* :All Metadata AND

medical OR medicine OR health* OR disease* OR illness* OR patient* OR hospital* OR disorder* : All Metadata
